# Renal Clearable Gold Nanoparticle-Functionalized Silk Film for *in vivo* Fluorescent Temperature Mapping

**DOI:** 10.3389/fchem.2020.00364

**Published:** 2020-05-15

**Authors:** Wei Hua, Yusheng Mao, Jinzhu Zhang, Lang Liu, Guolin Zhang, Shengyang Yang, Daniel Boyer, Chen Zhou, Fenfen Zheng, Shasha Sun, Shengling Lin

**Affiliations:** ^1^School of Environmental and Chemical Engineering, Jiangsu University of Science and Technology, Zhenjiang, China; ^2^Department of Chemistry and Chemical Engineering, Yangzhou University, Yangzhou, China; ^3^School of Natural Sciences, University of Central Missouri, Warrensburg, MO, United States

**Keywords:** gold nanoparticles, gold nanoparticles with silk film (AuNPs-SF), temperature sensing, implantable device, fluorescence imaging

## Abstract

Implantable optical sensing devices that can continuously monitor physiological temperature changes hold great potential toward applications in healthcare and medical field. Here, we present a conceptual foundation for the design of biocompatible temperature sensing device by integrating renal clearable luminescent gold nanoparticles (AuNPs) with silk film (AuNPs-SF). We found that the AuNPs display strong temperature dependence in both near-IR fluorescence intensity and lifetime over a large temperature range (10–60°C), with a fluorescence intensity sensitivity of 1.72%/°C and lifetime sensitivity of 0.09 μs/°C. When integrated, the AuNPs with biocompatible silk film are implanted in the dorsal region of mice. The fluorescence imaging of the AuNPs-SF in the body shows a linear relationship between the average fluorescence intensity and temperature. More importantly, <3.68% ID gold are left in the body, and no adverse effect is observed for 8 weeks. This AuNPs-SF can be potentially used as a flexible, biocompatible, and implantable sensing device for *in vivo* temperature mapping.

## Introduction

Implantable optical sensing devices (Dong et al., 2012; Ruckh and Clark, 2014) that are capable of reporting physiological information offer great opportunities for biomedical diagnosis, monitoring, and therapy (Balaconis and Clark, 2012; Brites et al., 2012). In particular, fluorescence-based sensing devices are of great interest due to their high spatial and temporal resolution, fast response, and ability to work in strong electromagnetic fields (Li et al., 2019). In this context, a large amount of research efforts have been devoted to hybrid inorganic fluorescent nanosensors such as carbon dots (Chandra and Singh, 2017; Khan et al., 2018), quantum dots (Zheng et al., 2015; Du et al., 2018), and gold nanoparticles (AuNPs) (Carattino et al., 2018; Fatino et al., 2018; Ma et al., 2018; Zhao et al., 2019) with biocompatible polymers (Zhou et al., 2009; Sun et al., 2016; Wu et al., 2019), hydrogel (Ming et al., 2016), or silk (Cheng et al., 2018) for implantable fluorescent sensing devices.

For hybrid implantable medical device (Ma et al., 2019), one of the key concerns is their toxicity and side effects after *in vivo* implantation (Ruckh and Clark, 2014). Indeed, many pioneer works were reported in the past decade for the exploration of biodegradable or biocompatible materials such as silk (Song et al., 2017; Qi et al., 2019), hydrogel, and polymers, leading to minimized toxicity from the substrate of an implanted device (Mieszawska et al., 2013). Nevertheless, little attention was paid to the fate of the integrated inorganic nanosensors in the hybrid implantable devices. In fact, inorganic nanoparticles (NPs) (Guo et al., 2019, 2020) pose severe threat *in vivo* because of their non-specific accumulation in the organelles and cells, as well as in the organs of reticuloendothelial system (RES). For example, more than 90% of the cadmium remained in the body after 90 days by intravenously injecting 50 nm of phospholipid-coated CdSe–CdS–ZnS quantum dots into monkeys (Chou and Chan, 2012), mainly accumulating in the kidney, liver, and spleen. Raghav et al. found that around 35% ID of AuNPs remained in the liver and spleen 120 days after administration (Goel et al., 2009), raising questions about the long-term side effects. To minimize the non-specific accumulation in the RES organs and the toxicity of inorganic NPs, several kinds of renal clearable inorganic NPs were developed (Zhou et al., 2011, 2012; Sun et al., 2016; Xue et al., 2020). For example, ~2 nm of glutathione-coated AuNPs can be efficiently cleared out through glomerular filtration within 24 h (more than 50%), with only (3.7 ± 1.9)% of the particles accumulated in the liver. For the CuS NPs with a hydrodynamic diameter of <6 nm, 95% are excreted intact through the renal urinary system within 24 h with minimal retention in the liver and spleen (Zhou et al., 2015).

**Figure 1:**
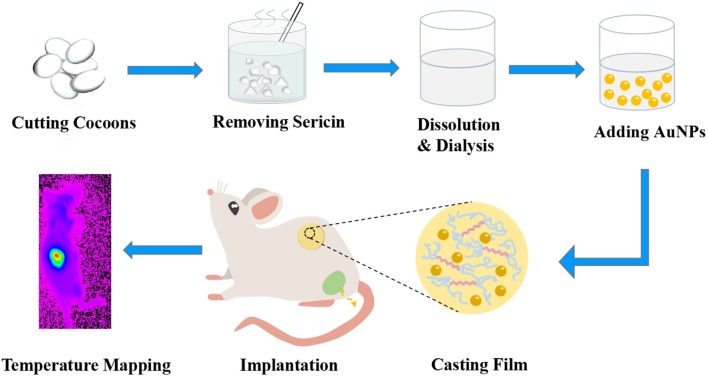
Schematic illustration of gold nanoparticles with silk film (AuNPs-SF) preparation, implantation, and temperature mapping.

While renal clearable NPs hold great potential in bioimaging with minimized toxicity (Liu et al., 2013, 2019; Zhou et al., 2015), whether these NPs can be utilized in hybrid implantable devices has not been explored in practice. Herein, we demonstrate a conceptual foundation of implantable temperature sensing device by integrating luminescent renal clearable AuNPs with biocompatible silk film (AuNPs-SF) (Figure 1). The AuNPs display a strong correlation among fluorescence intensity, lifetime, and temperature over all relevant physiological temperatures (10–60°C) in the near-IR region, with a fluorescence intensity sensitivity of 1.72%/°C and a lifetime sensitivity of 0.09 μs/°C. This property enables the AuNPs to serve as a temperature sensor in AuNPs-SF for physiological temperature sensing and imaging when implanted in the back of mice. More importantly, nearly all the AuNPs in the hybrid device could be cleared out of the body after 8 weeks of implantation, with only <3.68% ID Au remaining in the organs. These results indicate that the AuNPs-SF is promising to serve as implantable and wearable temperature sensing devices due to its superior sensitivity, biocompatibility, and processability.

## Materials and Methods

### Materials and Equipment

Gold chloride trihydrate (HAuCl_4_·3H_2_O), glutathione, ethanol, sodium carbonate (Na_2_CO_3_), calcium chloride (CaCl_2_), and glycerol were purchased from Aladdin Chemical Reagent Co., Ltd. All chemicals were used without further purification. Deionized (DI) water with the resistivity of ≥18.2 MΩ^*^cm was used throughout the experiments. *Bombyx mori* cocoons were obtained from the Sericulture Research Institute of Jiangsu University of Science and Technology as a gift. Female Balb/c mice were purchased from the Laboratory Animal Center of Jiangsu University.

High-resolution transmission electron microscopy (HRTEM) was performed on a JEM 2011(200 kV) transmission electron microscope (JEOL, Japan). The fluorescence spectra and lifetime were collected by an Edinburgh instrument spectrofluorometer FS5. *In vivo* fluorescence imaging was conducted by BRUKER Molecular Imaging. The gold content in organs was analyzed by an inductively coupled plasma mass spectrometry (ICP-MS XSERIES 2, Thermo).

### Preparation of Silk Solutions

The silk fibroin solution was produced according to the method reported previously. In brief, *Bombyx mori* cocoons were boiled in a 0.02 M sodium carbonate solution for 30 min to extract the silk fibroin protein and remove the sericin. The extracted silk was washed and dried for 12 h in a chemical hood. After drying, degummed silk cocoon was first dissolved in a ternary solvent system of CaCl_2_/H_2_O/EtOH solution (1:8:2 mole ratio) for 30 min at 85°C and dialyzed to remove salts in a cellulose tube (molecular cut-off, 12,000–14,000) against distilled water for 4 days at room temperature. The concentration was determined by measuring a volume of solution and the final dried weight.

### Synthesis of AuNPs

The NIR emitting AuNPs were synthesized according to the literature, and the purification method was modified. Briefly, 150 μl of 1 M HAuCl_4_ solution was added to 50 ml of 2.4 mM glutathione solution under vigorous stirring. The mixture was then heated at 90°C for 35 min. The resulting solution was cooled to room temperature and centrifuged at 21,000 × *g* to remove the large aggregates after the reaction. The supernatant was further purified by adjusting the pH of the solution to 3–4 and adding a small amount of ethanol into the aqueous solution (2:1, V_H2O_/V_ethanol_), followed by centrifuging the solution at 4,000 × *g* for 5 min to discard the supernatant. The precipitates were then suspended in 300 μl of PBS buffer. This as-prepared AuNPs were stored at 4°C for further use.

### Synthesis of AuNPs-SF

The concentration of purified AuNPs was 6.75 × 10^−5^ mol/L. Two hundred microliters of AuNPs was added to 2 ml of silk fibroin solution with 2–3% glycerol. The mixed solution was cast in polyethylene disk and dried at 30°C for 5 h. After drying, the obtained AuNPs-SF is 0.067 g. The amount of AuNPs in the film is around 2.6% (w/w).

### Animal Experiments

Female Balb/c mice (6–8 weeks old, three in each group) were anesthetized with an intraperitoneal injection of 10% chloral hydrate anesthetic. After the mice were anesthetized, the backs of the mice were depilated. Once the animal was lightly anesthetized, hair was removed from the back of the three mice. A small longitudinal incision was made through the skin, and the sterile implants (ultraviolet sterilization) were inserted. The incision was closed with a Dexon 5–0 suture. The animals were monitored as soon as the surgery was completed until ambulatory. *In vivo* imaging was performed on the mice to observe the changes in body temperature as their body temperature increased and decreased. After 60 days, the mice were euthanized by carbon dioxide euthanasia, and the organs were taken. The isolated tissues/organs with known weights were completely lysed in 2 ml of freshly made aqua regia in screw capped glass bottles (5 ml) separately for 1 week. After ultrasonication for 20 min, 1,000 × *g* was centrifuged for 5 min to remove the precipitate. Then the aqua regia was completely evaporated for ICP analysis.

### Ethics Statement

This study was carried out in accordance with the principles of the Basel Declaration and recommendations of the Guide for the Care and Use of Laboratory Animals, Laboratory Animal Management Committee of Jiangsu University. The protocol was approved by the Laboratory Animal Management Committee of Jiangsu University.

## Result and Discussion

**Figure 2:**
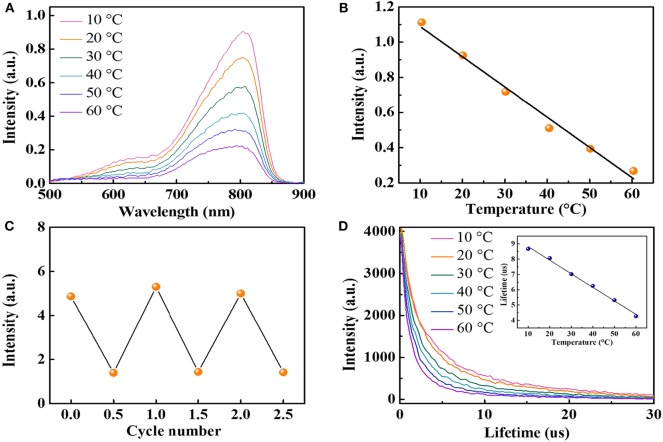
**(A)** The fluorescence spectra of AuNPs at different temperatures (excited at 406 nm). **(B)** The relationship between fluorescence intensity at 800 nm and temperature in the range of 10 to 60°C. **(C)** Temperature sensing reversibility of AuNPs. **(D)** Fluorescence decay curves of AuNPs (inset: average lifetime at various temperatures from 10 to 60°C).

The present near-IR fluorescent temperature sensor is the glutathione-coated gold nanoparticles (AuNPs) with a luminescent emission around 800 nm. AuNPs were synthesized with a method reported previously (Zhou et al., 2011). High-resolution transmission electron microscopy showed that the average diameter of the AuNPs is around 2.6 ± 0.3 nm (Figure S1). Figure 2A shows the temperature dependence of the fluorescence intensity of AuNPs. A 4-fold emission intensity decrease is observed over the temperature range of 10 to 60°C, indicating high-temperature sensitivity of the AuNPs. The fluorescence intensity at 800 nm is linearly dependent on the temperature with a correlation coefficient of 0.985, and the fluorescence intensity decreased by 1.72%/°C when the temperature is increased from 10 to 60°C (Figure 2B), which is comparable with the reported upconversion nanoparticles (Liu et al., 2013, 2019), indicating superior high sensitivity of AuNPs. A similar linear relationship was observed when the temperature decreased from 60 to 10°C (Figure S2). Notably, different from QDs with a remarkable temperature-dependent spectra shift (Maestro et al., 2010; Yang et al., 2011; Huang et al., 2016), the emission spectra of AuNPs did not shift within the investigated temperature window, making it suitable for *in vivo* temperature mapping. The heating–cooling cycling experiment shows the temperature-dependent fluorescence intensity demonstrates good reversibility and reproducibility between 10 and 60°C (Figure 2C). The wide linear temperature detection range and good reversibility reveal the feasible applications of AuNPs for the monitoring of physiological temperature.

Fluorescence lifetime is known to be more reliable than intensity-based measurements because the fluorescence intensity may suffer from a variation of the sensor concentration and drifts of the optoelectronic system (lamps and detectors) (Peng et al., 2010). Therefore, we also measured the fluorescence lifetime of AuNPs at different temperatures. AuNPs display a fluorescence decay that can be well-fitted with two exponentials, with a short lifetime (1.1 μs < τ_1_ <2.08 μs) and a long lifetime (7.63 μs < τ_2_ <11.4 μs). Figure 2D shows the relationship between the average fluorescence lifetime of AuNPs and temperatures. Detail analysis suggests a linear dependency (*R*^2^ = 0.995) of the average fluorescence lifetime and temperature. The average lifetime of AuNPs decreases from 8.68 μs at 10°C to 4.28 μs at 60°C (Figure 2D, inset), correlating to a sensitivity of 0.09 μs/°C. Similar to the fluorescence intensity, the lifetime change of AuNPs is also reversible. When the temperature decreases, the fluorescence lifetime returns to the initial value (Figure S3). Considering the luminescence of AuNPs is induced by the charge transfer from glutathione to Au(I) (Zheng et al., 2012), the high temperature will accelerate the charge transfer and, thus, decrease the lifetime. It should be noted that the lifetime of AuNPs is in the microsecond range, which is three orders higher than the lifetime of the biological tissues. Therefore, the AuNPs in biological samples can avoid the autofluorescence background from the tissue (Shang et al., 2013).

**Figure 3:**
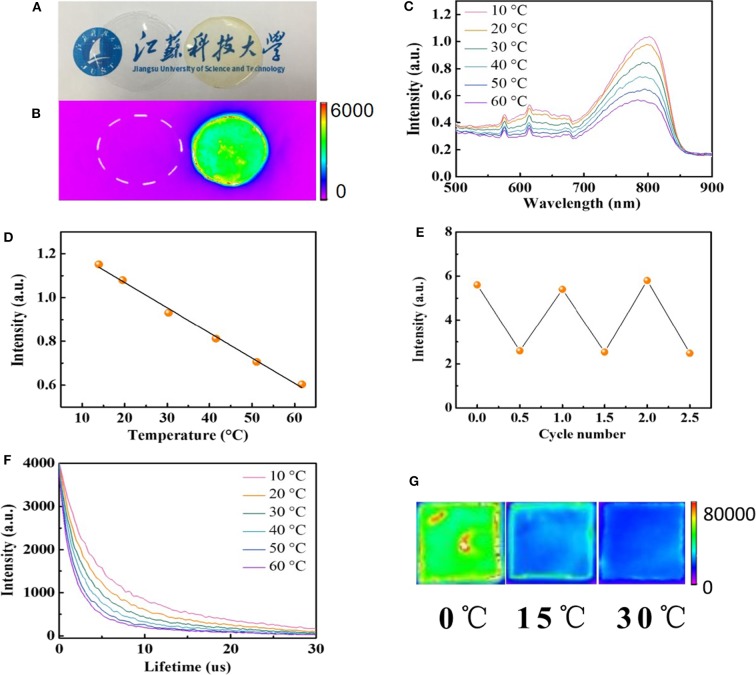
**(A)** Photographs of silk fibroin film under ambient light (left: pure silk fibroin film; right: AuNPs-SF). **(B)** Photographs of silk fibroin films in the fluorescent imaging system (left: pure silk fibroin film; right: AuNPs-SF). **(C)** The fluorescence spectra of AuNPs-SF at different temperatures (excited at 406 nm). **(D)** The relationship between the fluorescence intensity at 800 nm and the temperature in the range of 10 to 60°C. **(E)** Temperature sensing reversibility of AuNPs-SF. **(F)** Luminescence decay of AuNPs-SF. **(G)** Near-IR fluorescence photograph of the AuNPs-SF at different temperatures.

To measure the spatial and temporal temperature of the body, the temperature sensor is generally attached to the freely curved surface of the body (Nakata et al., 2017; Ota et al., 2017). In our system, we chose a silk film to integrate temperature-sensitive AuNPs (AuNPs-SF) as the flexible temperature sensor. The AuNPs-SF was prepared via a regenerated silk fibroin process. By adding glycerol during the synthesis process, the silk film can be stable in aqueous solution without dissolution for several years, which allows the film for long-term *in vivo* temperature monitoring. Glycerol works as a cross-linker, which can form hydrogen bonds with hydrophilic polar groups in silk fibroin and induces high crystallinity of the silk fibroin membrane (Lu et al., 2009, 2011; Brown et al., 2016). Both the pure silk film and AuNPs-SF are flexible and transparent, but the AuNPs-SF displays a pale-yellow color due to the absorption from the incorporated AuNPs (Figure 3A). No fluorescence can be observed from pure silk film, but the AuNPs-SF shows strong fluorescence in the near-IR window, indicating that the fluorescence property of AuNPs remained after the silk film fabrication process (Figure 3B). To test the film stability, AuNPs-SF was incubated in PBS buffer for 4 weeks. The film was still intact, and no fluorescence was observed from the PBS buffer (Figure S4A), suggesting a stable fixation of AuNPs in the insoluble silk matrix in the *ex vivo* environment. Figure 3C and Figure S4B show the fluorescence spectra of the hybrid films collected at various temperatures between 10 and 60°C. The fluorescence intensity of AuNPs-SF decreases with an increment of temperature (Figure 3C). The fluorescence intensity at 800 nm changes linearly with temperature (Figure 3D). A similar linear relationship was observed when the temperature decreases from 60 to 10°C (Figure S4C). A 2-fold fluorescence intensity was observed from AuNPs-SF from 10 to 60°C. The lower intensity response of AuNPs-SF compared with that of the AuNPs in PBS can be attributed to the free motion of AuNPs that was restricted via the incorporation into the silk film. To evaluate the durability of the AuNPs-SF, a continuous response test was performed in the physiological temperature range of 30 to 42°C. The AuNPs-SF still demonstrates a very high degree of temperature fitting (Figure S5). Such high accuracy in the physiological range is beneficial for *in vivo* temperature mapping and future clinical applications. The temperature-dependent fluorescence of AuNPs-SF is highly reversible upon temperature cycling, as shown for three complete cycles between 10 and 60°C (Figure 3E). In addition, the lifetime of AuNPs-SF is also temperature dependent with a linear relationship (Figure 3F and Figure S6). The AuNPs-SF displays a fluorescence decay with a short lifetime (1.62 μs < τ_1_ <2.73 μs) and a long lifetime (9.12 μs < τ_2_ <12.82 μs). The promoted lifetime of AuNPs-SF compared to that of AuNPs can be attributed to the inhibited non-radiative transition when AuNPs were confined in the silk film (Tian et al., 2015). Real-time temperature mapping is demonstrated with a fluorescence imaging system. Figure 3G shows the near-IR fluorescence images of the sensor film at 0, 15, and 30°C, respectively.

**Figure 4:**
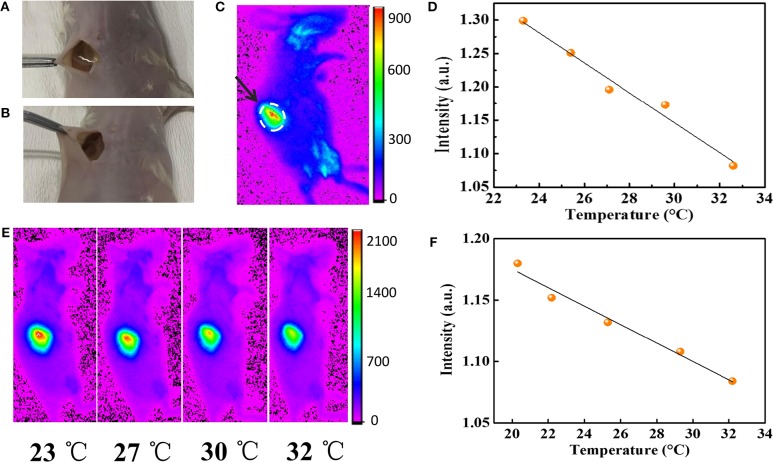
**(A)** The implantation of AuNPs-SF in the dorsal region of a mouse. **(B)** The AuNPs-SF can perfectly fit with the body. **(C)** *In vivo* fluorescence imaging of balb/c mouse. **(D)** Relationship between mean fluorescence intensity and temperature. **(E)** *In vivo* fluorescence imaging of a live mouse in anesthesia state. From left to right, temperature from 23 to 32°C (λ_ex_ = 410 nm, λ_em_ = 830 nm). **(F)** Relationship between mean fluorescence intensity and temperature of implanted AuNPs-SF after 10 days.

To demonstrate the application of AuNPs-SF in the mapping of physiological temperature, the AuNPs-SF was implanted into the dorsal region of female balb/c mice (Figure 4A), and the temperature is monitored by a fluorescence imaging system. The high flexibility of the film enables it to fit well with the mice tissue after implantation (Figure 4B). Owing to the strong emission in the near-IR range, the film can be easily distinguished from the normal tissue (Figure 4C). The *in vivo* fluorescence imaging of the film shows a linear relationship between the fluorescence intensity and temperature in the physiological range from 23 to 32°C (Figures 4D,E). Limited by the duration of anesthesia and the awakening of the mice with the increase in temperature, the body temperature was monitored for 23–32°C. While part of the AuNPs can be released from the silk film within the physiological environment, a linear relationship between the fluorescence intensity and temperature (*R*^2^ = 0.973) can be still observed when the mice is imaged after a 10 day implantation (Figure 4F).

We also explored the long-term toxicity and side effect of AuNPs-SF in mice. The mice were continuously monitored for 8 weeks, and no adverse effect was observed from the implanted film. After 8 weeks, little fluorescence signal can be seen in other parts of the mice, implying no significant accumulation of AuNPs in the superficial layer tissues. The biodistribution of AuNPs in the mice was studied by inductively coupled plasma mass spectrometry (ICP-MS). The results show that only 4.220 ± 1.425% ID of AuNPs remained in the silk film substrate. Only <3.68% ID gold were found in the organs, from which 1.599 ± 0.432% ID was found in the liver (Table S1). The absence of the other 92.1% Au in the implanted AuNPs-SF and the mice body suggests an Au elimination through the renal urinary system. This result is consistent with previous report that glutathione-coated AuNPs are renal clearable with very low accumulation in RES organs (Zhou et al., 2011, 2012).

## Conclusions

In conclusion, we developed a hybrid AuNPs-SF as implantable fluorescent temperature sensor for continuous *in vivo* temperature sensing using the temperature-dependent fluorescence trait of AuNPs. By implanting the sensor film to the mice, the film can be utilized for continuous mapping of temperature distributions in tissues. More importantly, the AuNPs can be efficiently cleared out from the body due to their renal clearance property, with only <3.68% ID gold left in the body after 8 weeks, significantly minimizing the potential toxicity and side effects caused by the implantable sensing devices. Overall, the use of fluorescence-based temperature sensor holds great promise for the continuous *in vivo* temperature monitoring, allowing wireless signal transition and long-lasting functionality. This work achieves an important step for the future development of implantable and wearable devices with inorganic sensors that are ultra-sensitive, implant biocompatible, and innoxious.

## Data Availability Statement

All datasets generated for this study are included in the article/supplementary material.

## Ethics Statement

The animal study was reviewed and approved by Laboratory Animal Management Committee of Jiangsu University.

## Author Contributions

WH and SS completed the majority of the research work. YM, LL, and GZ helped with the synthesis of AuNPs and silk films. JZ helped with the fluorescence and lifetime measurement and analysis. DB helped with synthesis of AuNPs. FZ helped with the animal study. SY, CZ, and SL reviewed, analyzed, and interpreted the data. WH, SS, and SL wrote the paper. All authors discussed the results and commented on the manuscript.

## Conflict of Interest

The authors declare that the research was conducted in the absence of any commercial or financial relationships that could be construed as a potential conflict of interest.
