# Supplementary material for: Renal Clearable Gold Nanoparticle-Functionalized Silk Film for in vivo Fluorescent Temperature Mapping
Source: Front Chem. 2020 May 15;8:364. doi: 10.3389/fchem.2020.00364 (PMC7243850; doi:10.3389/fchem.2020.00364)
Supplement: Supplementary file 1 [file Table_1.DOCX]

Supplementary Material

# Supplementary Figures and Tables


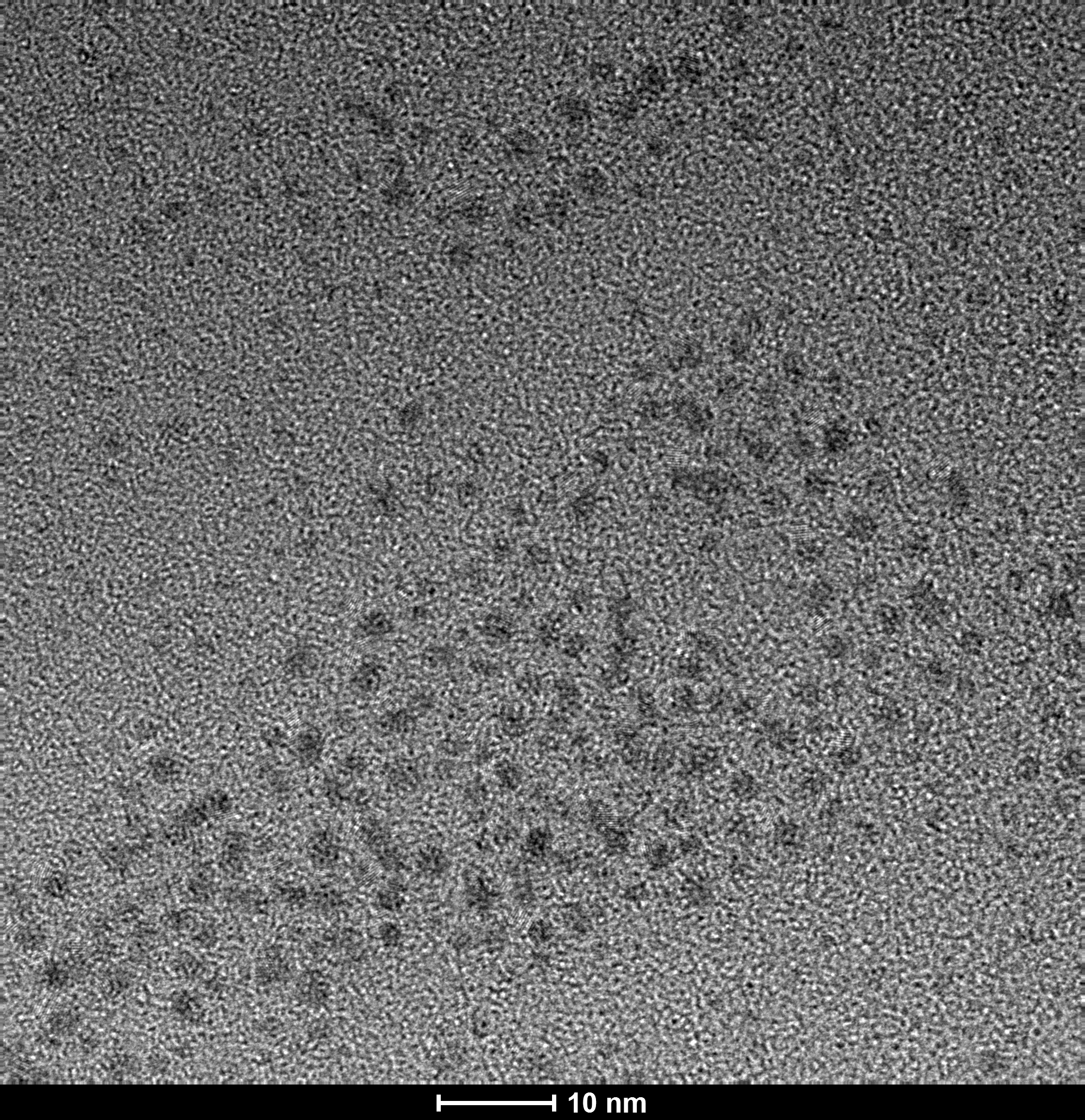


**Figure S 1.** High-resolution transmission electron microscopy of the AuNPs.


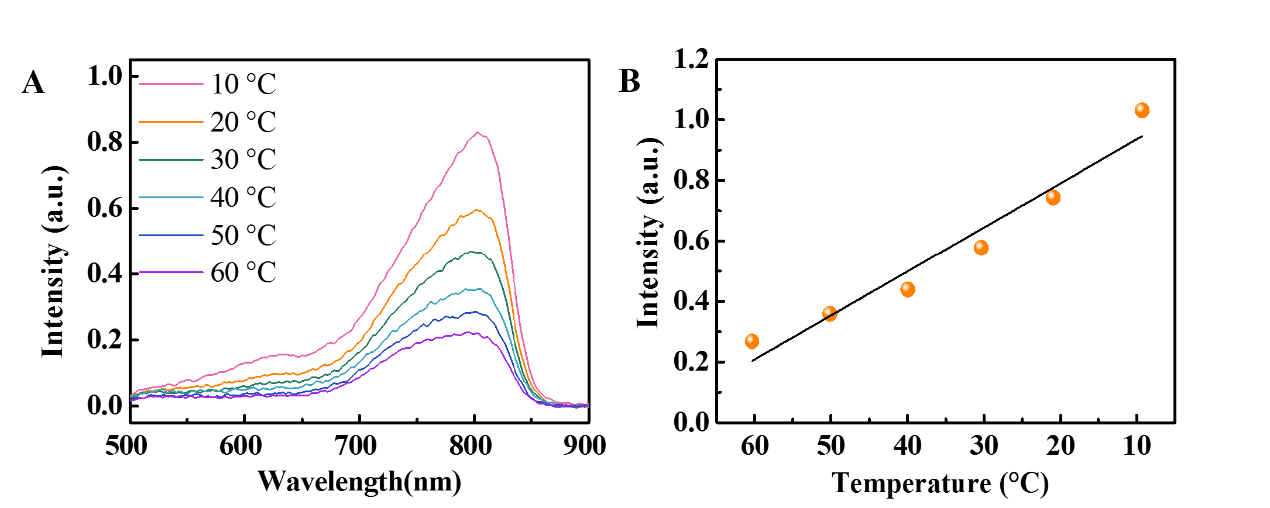


**Figure S 2.** **(A)** The fluorescence intensity of AuNPs varies with the decrease of temperature. **(B)** The fluorescence intensity at 800 nm in the range of 60 to 10 °C.


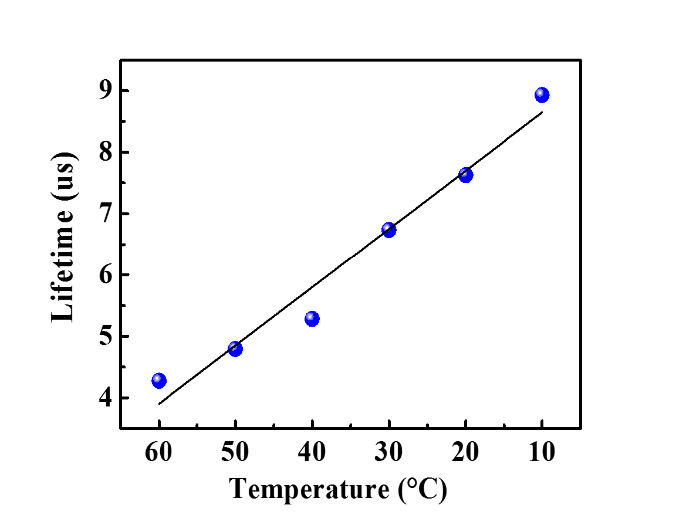


**Figure S 3.** The lifetime of AuNPs at various temperatures from 60 to 10 °C.


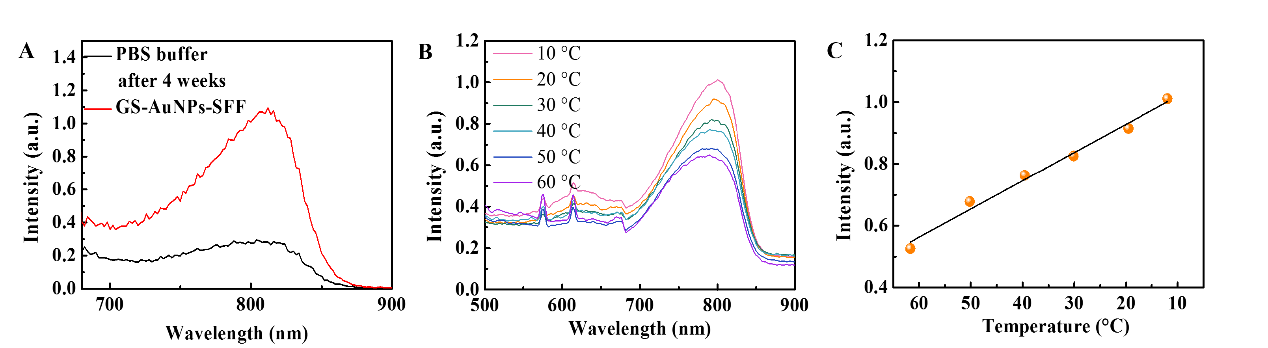


**Figure S 4.** Fluorescence spectroscopy. **(A)** The AuNPs-SF was put into PBS buffer solution for four weeks, removed and tested separately. The black curve is PBS buffer solution, and the red curve is AuNPs-SF. **(B)** The fluorescence intensity of AuNPs-SF varies with the decrease of temperature. **(C)** Temperature sensitivity of AuNPs-SF between 60 and 10 °C.


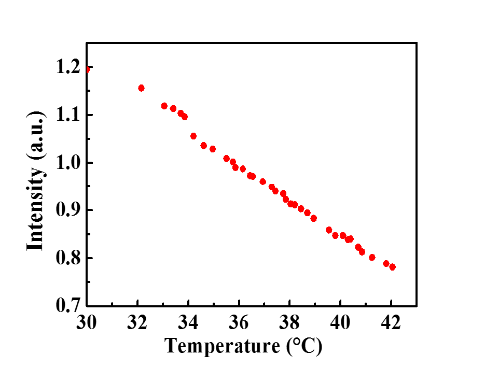


**Figure S 5.** Determination of continuous fluorescence intensity from 30 to 42 °C.


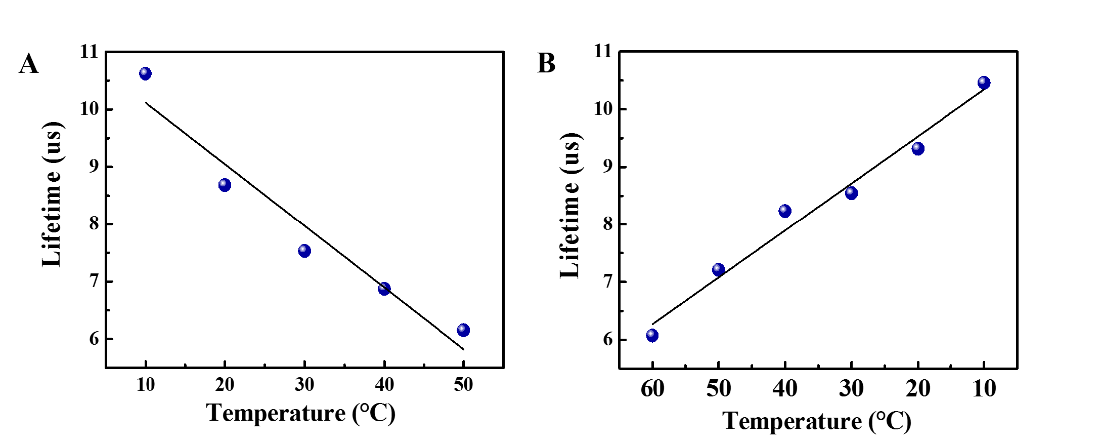


**Figure S 6.** **(A)** Lifetime of AuNPs-SF at various temperatures from 10 to 50 °C. **(B)** Lifetime of AuNPs-SF at various temperatures from 60 to 10 °C.

| Organ | (%ID) | Organ | (%ID) |
| --- | --- | --- | --- |
| Heart | 0.331 ± 0.234 | Large intestine | 0.285 ± 0.0476 |
| Liver | 1.599 ± 0.432 | Small intestine | 0.395 ± 0.100 |
| Spleen | 0.246 ± 0.111 | Fat | 0.077 ± 0.015 |
| Stomach | 0.133 ± 0.047 | Skin | 0.109 ± 0.015 |
| Kidney | 0.270 ± 0.066 | Muscle | 0.085 ± 0.009 |
| Lung | 0.152 ± 0.065 | Film | 4.220 ± 1.425 |

**Table S 1.** The biodistribution of AuNPs was summarized after 60 days of implantation.
